# The child survival impact of the Ghana Essential Health Interventions Program: A health systems strengthening plausibility trial in Northern Ghana

**DOI:** 10.1371/journal.pone.0218025

**Published:** 2019-06-12

**Authors:** Ayaga A. Bawah, John Koku Awoonor-Williams, Patrick O. Asuming, Elizabeth F. Jackson, Christopher B. Boyer, Edmund W. Kanmiki, Sebastian F. Achana, James Akazili, James F. Phillips

**Affiliations:** 1 Regional Institute for Population Studies, University of Ghana, Legon, Accra, Ghana; 2 Policy, Planning, Monitoring and Evaluation Division, Ghana Health Service, Accra, Ghana; 3 Business School, University of Ghana, Legon, Accra; 4 Heilbrunn Department of Population and Family Health, Mailman School of Public Health, Columbia University, New York, New York, United States of America; 5 Harvard School of Public Health, Boston, Massachusetts, United States of America; 6 Navrongo Health Research Centre, Ghana Health Service, Navrongo, Upper East Region, Ghana; Cincinnati Children's Hospital Medical Center, UNITED STATES

## Abstract

**Background:**

The Ghana Health Service in collaboration with partner institutions implemented a five-year primary health systems strengthening program known as the Ghana Essential Health Intervention Program (GEHIP). GEHIP was a plausibility trial implemented in an impoverished region of northern Ghana around the World Health Organizations (WHO) six pillars combined with community engagement, leadership development and grassroots political support, the program organized a program of training and action focused on strategies for saving newborn lives and community-engaged emergency referral services. This paper analyzes the effect of the GEHIP program on child survival.

**Methods:**

Birth history data assembled from baseline and endline surveys are used to assess the hazard of child mortality in GEHIP treatment and comparison areas prior to and after the start of treatment. Difference-in-differences (DiD) methods are used to compare mortality change over time among children exposed to GEHIP relative to children in the comparison area over the same time period. Models test the hypothesis that a package of systems strengthening activities improved childhood survival. Models adjusted for the potentially confounding effects of baseline differentials, secular mortality trends, household characteristics such as relative wealth and parental educational attainment, and geographic accessibility of clinical care.

**Results:**

The GEHIP combination of health systems strengthening activities reduced neonatal mortality by approximately one half (HR = 0.52, 95% CI = 0.28,0.98, p = 0.045). There was a null incremental effect of GEHIP on mortality of post-neonate infants (from 1 to 12 months old) (HR = 0.72; 95% CI = 0.30,1.79; p = 0.480) and post-infants (from 1 year to 5 years old) -(HR = 1.02; 95% CI = 0.55–1.90; p = 0.940). Age-specific analyses show that impact was concentrated among neonates. However, effect ratios for post-infancy were inefficiently assessed owing to extensive survival history censoring for the later months of childhood. Children were observed only rarely for periods over 40 months of age.

**Conclusion:**

GEHIP results show that a comprehensive approach to newborn care is feasible, if care is augmented by community-based nurses. It supports the assertion that if appropriate mechanisms are put in place to enable the various pillars of the health system as espoused by WHO in rural impoverished settings where childhood mortality is high, it could lead to accelerated reductions in mortality thereby increasing survival of children. Policy implications of the pronounced neonatal effect of GEHIP merit national review for possible scale-up.

## Introduction

In recent decades, Ghana has been at the forefront of developing community-based primary health care. Policies that can be traced to the Alma Ata accord [1], refined and tested by an experimental trial of the Navrongo Health Research Centre (NHRC) [2–4], and replicated at scale by the national program determined that community-based primary health care in rural Ghana can save childhood lives and reduce fertility [5, 6]. In response to this evidence, the Ghana Health Service (GHS) adopted a policy known as Community-based Health Planning and Services (CHPS) in 1999 [7]. Implementation aimed to scale-up lessons from the Navrongo trial [8] by deploying certified community nurses to community locations, organizing community support for their work, and procuring essential technology, supplies, and equipment to support service delivery work. CHPS health posts termed *Community Health Compounds* were developed in service catchment zones where nurses would live and work [9]. Each CHPS nurse was provided with at least 18 months of training in primary health care services, with an additional six months of practical internship training. Nurses were supported by community volunteers who have varying degrees of training and responsibilities but are usually assigned health promotional tasks that backstop curative and preventive health service activities. Monitoring of the Navrongo project showed that posting nurses to community locations reduced childhood mortality by over half in only three years [6], a finding that was successfully replicated in a series of small scale implementation research projects [10, 11].

Despite this promising evidence, a variety of service delivery, manpower, communication, logistics, resource management, and leadership bottlenecks have constrained the pace of CHPS scale up [12, 13]. Moreover, proven interventions have yet to be introduced into the CHPS program. As of 2008, CHPS had reached only 8% of the population. To address these bottlenecks, an embedded implementation science program known as the Ghana Essential Health Interventions Program (GEHIP) was launched in 2010 in the Upper East region of Ghana to test the hypothesis that a novel set of interventions aimed at strengthening primary health care by developing leadership, trainings, information for decision making, logistics and health worker deployment would accelerate the scale-up of CHPS functioning and impact on child mortality [14, 15]. The goal of this paper is to a) examine the under-five child survival in both treatment and control areas of the GEHIP program. b) Assess the effect of GEHIP on under-five mortality (including neonatal & infants) and to c) determine the factors associated with under-five mortality.

### The Ghana Essential Health Interventions Program (GEHIP)

In 2009, the Ministry of Health (MoH) convened a panel of experts to clarify operational factors that explained why CHPS was proceeding so slowly [16]. The expert team interviewed district managers, sub-district supervisors, and frontline workers about their perception of community health service systems development problems and needs. District management teams involved in the review were purposefully selected to ensure that both rapid implementation districts and poorly performing districts were included in the appraisal. Recommendations elicited by this process were assembled into a set of posited actions that could be taken by district managers to accelerate CHPS scale-up. This included a set of interventions designed to address district management reluctance to proceed with CHPS implementation. Shortage of nursing staff was not the problem. Throughout Ghana, nurses had been recruited and trained to provide community health care, but most villages lacked health posts where these nurses could be posted. Strategies for addressing the revenue requirements of constructing health posts were not widely understood. Where CHPS had been rapidly implemented, managers had developed community engagement strategies that led to low cost volunteer construction of community health posts. This permitted managers to launch CHPS in a few such communities. Pilot implementation could be used to demonstrate the popularity of CHPS service, leading to grassroots political support that could catalyze District Assembly commitment to financing CHPS start-up costs.

As noted elsewhere, GEHIP’s interventions sought to address the key challenges of the health system as identified by the MoH team of experts. The main interventions focused on strengthening primary health care by developing leadership skills at the district and sub-district levels, trainings of frontline workers to deliver critical care at the community level, developing an information management system to support decision-making at the operational level, developing strategies for supplies and logistics management and finally developing a sustaining emergency referral system for mothers and newborn care. We believe that assembling these interventions into coordinated package of activities would lead to improvements in childhood survival. Of particular importance to GEHIP strategies and action were frameworks for health system strengthening that emphasized the importance of developing district leadership capabilities as interacting essential “pillars” of effective system functioning [17, 18]. By focusing on developing leadership, information for decision-making, budgeting, logistics, training, and worker deployment, the provision of health services at community locations could be enhanced, with measurable impact on the survival of children.

In conjunction with these systems strengthening interventions, elements of GEHIP focused on adding primary health care components that were lacking in the program. Sets of health systems strengthening activities were pursued involving community-engagement for organizing the provision of the WHO recommended regimen for integrated management of childhood illness [19]. Particular attention was directed to addressing the absence of emergency public health services. Frontline workers had been poorly trained and inadequately equipped to deal with the lead causes of neonatal morbidity and mortality. In response to this deficiency, a comprehensive referral service was developed for GEHIP districts that involved the promotion of facility based delivery, consignment of low cost ambulances, the training and deployment of volunteer drivers, the organization of a communication system, and a process of convening community engagement for sustaining social support for referral operations [14, 20]. Most critical to newborn health, however, was a region-wide program to promote worker compliance with WHO recommended procedures [21] for managing neonatal asphyxia [22, 23], septicemia [24–26], acute respiratory infections [27–29] and malaria [30].

**Fig 1:**
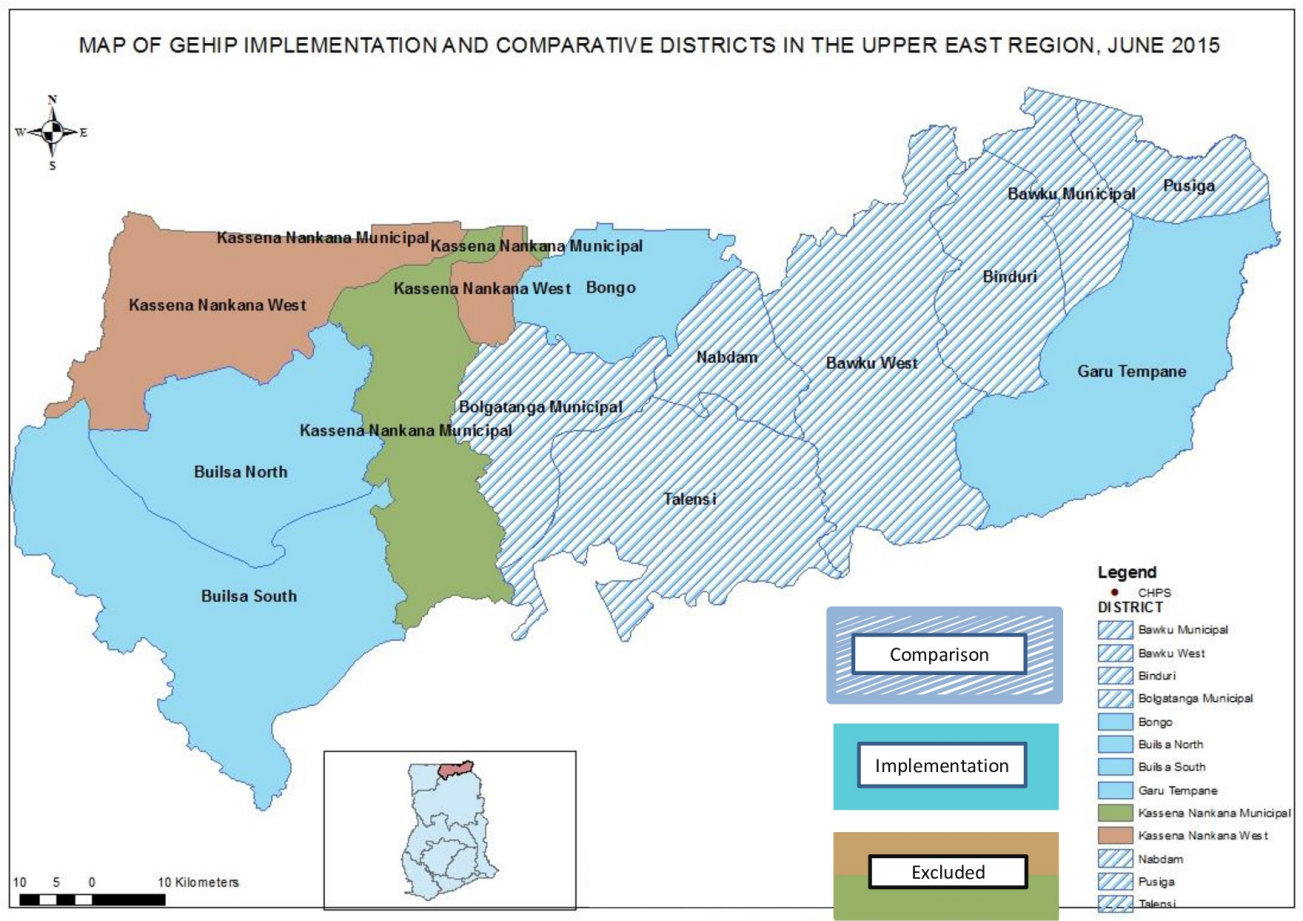
Map of the GEHIP implementing and comparison districts in the Upper East Region.

While worker training was implemented in all UER districts, GEHIP district interventions were designed to test ways to improve program access to WHO recommended modalities and procedures by implementing a trial package of leadership, community engagement, and emergency health service interventions. Commencing in 2010 as a plausibility study in a subset of four Upper East Region (UER) districts, the trial was known as the Ghana Essential Health Interventions Program (GEHIP) [14, 31]. Seven neighboring UER districts comprised a comparison area where GEHIP interventions would not be introduced. The combined population of the districts of Ghana’s Upper East Region (UER) was estimated to be 1.1 million at the onset of GEHIP [32, 33]. All major GEHIP program components were underway by July of 2011, and observation extended for 3.5 years, ending in early 2015. Two of the 13 UER districts were excluded from GEHIP because research programs of the NHRC in these districts produced atypical demographic and health conditions (Fig 1).

## Methods and materials

GEHIP was convened to test the hypothesis that health systems strengthening at the district level causes childhood mortality decline. Testing this hypothesis required longitudinal observation of organizational change and linked data on parental health seeking behavior and childhood mortality outcomes. Survey research was applied with cluster sampling to gauge changing access to health facilities over time, due to the GEHIP focus on expanding CHPS coverage. A baseline cluster survey was repeated at the endline, providing for the longitudinal documentation of expanding service operations by linking information on proximity to hospitals and clinics with monitoring data recording changes in the coverage of CHPS. Since impact of health care varies by age, the analysis took into consideration the age of the child, as well as ways in which the system at each level was changing relative to the exact age of each sample child as time progressed.

### The context

GEHIP was initially implemented in three districts of the UER: Builsa, Bongo, and Garu-Tempane (Fig 1). Seven other UER districts served as a project comparison area. At the onset of the project, Builsa was split by an act of Parliament into two districts (Builsa North and South), making four treatment districts, in all.(see Fig 1).

The 11 project districts rank among the poorest 5% of Ghana’s districts, each with economies that are dominated by subsistence agriculture. According to the Ghana Statistical Service (GSS), per capita income for these districts is about a quarter that of Ghana, ranking equivalently with the districts of the Upper West Region as the two most impoverished regions of Ghana [33]. Against this backdrop of profound economic adversity, the region is also health service deprived. Although Bongo and Builsa-North have hospitals, other districts in the region rely upon fragile and incomplete referral services or upgraded sub-district health centers for hospital care. There is a regional hospital in Bolgatanga, but apart from obstetrical care, specialized medical care of any kind is not available in the UER.

Where the UER has registered progress, however, is with its implementation of community-based primary health care. Where coverage of the program has been lacking, interim facilities are often available, a strategy that has become more prominent in the GEHIP era. Thus, while tertiary health care is poorly developed, community-based primary health care has become more accessible in recent years, providing access to basic curative and preventive health services for children.

Birth histories and corresponding information about deaths among children ever born were collected during the interviews of all women resident in sample households aged 15 to 49. Baseline survey interviews of 5511 women of reproductive age, out of an estimated sample of 6000, yielding an achieved sample of 91.8 include survival histories of 7410 children ever born who were ever 60 months of age or less during the five year period prior to each survey. Correspondingly, 5914 out of a targeted sample of 7588 women were interviewed in the endline, yielding a 76% achieved sample with survival histories of 7044 children ever born who were ever 60 months of age or less five years prior to the survey. Sampling was performed using a two-stage cluster design. In the first stage for the baseline, 66 clusters were apportioned among district census enumeration areas proportional to size using population projections based on the 2010 population and housing survey [34, 35]. In the second stage, random household selection proceeded within each cluster proportional to enumeration area size until the target sample total of 6000 women of reproductive age were selected. At the endline, the baseline surveys were reused to establish longitudinality of GEHIP exposure observation. However, since relisting and stage two resampling was pursued, GEHIP is a panel at the cluster level only. Interviews were conducted in the prevailing local language of sample households.

For the purposes of the study, a live birth was defined as one in which the child cried or showed signs of life at birth such as pulsation of the umbilical cord or definite muscle movement. Crude annual estimates of under-5 mortality were calculated and compared to national estimates from the Ghana DHS over the same period [36–39]. Childhood survival was assessed for all children under 60 months of age. Observation of children was censored at 60 months of age or by the survey date. Analysis time was age of life in months.

Since the mortality hazard followed a different pattern for neonates, post-neonate infants, and post-infants, the proportional hazard assumption was violated. This was addressed by introducing a categorical time interaction into our model to provide separate mortality hazard ratio estimates for (1) neonates in the first one month of life, (2) post-neonatal infants from age one month through 11 months of age, and (3) post-infant children from 12 months through 59 months of age. Although a separate hazard function was estimated for neonates, defined in days of age, results were identical to those produced by the age in month model.

Covariates arrayed in Table 1 were incorporated in the full model [40–42]. Maternal variables included mother's age at birth, religion, literacy, occupation, parity, wealth, marital status, and polygamy. Childhood characteristics included birth order, sex, and gestational age. Analyses incorporate an estimate of access to hospitals or sub-district health centers by measuring household distance to the nearest such facility via global positioning methods. Household wealth quintiles were constructed using principal components analysis (PCA) of discrete asset indicators [43] that defined access to sanitation and water, household possession of consumer durables (bicycle, radio, bicycle, motorbike, etc), and dwelling unit construction. Since these indicators were discrete variables, polychoric correlation matrix analysis was applied [44]. The principal component explained 40.9% of the common variance.

**Table 1:** Balance of study variables across treatments, Upper East Region, Ghana, for all children under five years of age during the five year period prior to each survey (2005–2014), adjusted for the effects of cluster sampling.

Covariates	Baseline, 2010			Endline, 2015		
	control	Intervention	p-value	control	Intervention	p-value
	(n = 3,705)	(n = 3,705)		(n = 3,409)	(n = 3,635)	
Gender:			0.117			0.018
Male	1854 (50.6%)	1940 (53.0%)		1709 (50.3%)	1928 (53.1%)	
Female	1812 (49.4%)	1721 (47.0%)		1689 (49.7%)	1704 (46.9%)	
Birth type:			0.012			0.944
Singleton	3541 (95.6%)	3602 (97.2%)		3311 (97.1%)	3529 (97.1%)	
Multiple	164 (4.4%)	103 (2.8%)		98 (2.9%)	106 (2.9%)	
Gestation			0.008			0.011
9 months	3668 (99.0%)	3629 (97.9%)		3375 (99.0%)	3564 (98.0%)	
< 9 months	37 (1.0%)	76 (2.1%)		34 (1.0%)	71 (2.0%)	
Birth spacing			0.609			0.462
≥ 24 months	2751 (74.3%)	2769 (74.7%)		3119 (91.5%)	3347 (92.1%)	
< 24 months	954 (25.7%)	936 (25.3%)		290 (8.5%)	288 (7.9%)	
Parity			0.195			0.359
Nulliparous	653 (17.6%)	596 (16.1%)		767 (22.5%)	745 (20.5%)	
Primipara	645 (17.4%)	604 (16.3%)		656 (19.2%)	676 (18.6%)	
Multipara	1670 (45.1%)	1625 (43.9%)		1561 (45.8%)	1704 (46.9%)	
grand multipara	737 (19.9%)	880 (23.8%)		425 (12.5%)	510 (14.0%)	
Maternal Age			0.909			0.092
15–20	450 (12.4%)	462 (12.7%)		366 (10.7%)	325 (8.9%)	
20–34	2555 (70.5%)	2544 (69.8%)		2411 (70.7%)	2527 (69.5%)	
35–49	621 (17.1%)	641 (17.6%)		632 (18.5%)	783 (21.5%)	
Maternal marital status:			0.343			0.587
Unmarried	1054 (29.0%)	904 (24.6%)		374 (11.0%)	426 (11.7%)	
other wives	948 (26.1%)	1132 (30.8%)		1019 (29.9%)	1194 (32.9%)	
Monogamous	1632 (44.9%)	1640 (44.6%)		2013 (59.1%)	2013 (55.4%)	
Maternal literacy			0.109			0.791
No	3354 (90.5%)	3189 (86.1%)		2935 (86.1%)	3156 (86.8%)	
Yes	351 (9.5%)	516 (13.9%)		474 (13.9%)	479 (13.2%)	
Maternal education			0.188			0.317
Less than secondary	3636 (98.2%)	3592 (97.0%)		3299 (96.8%)	3467 (95.4%)	
Secondary or higher	67 (1.8%)	112 (3.0%)		110 (3.2%)	168 (4.6%)	
Wealth (household)			0.064			0.007
Less Poor (Q2—Q5)	2501 (67.6%)	2159 (58.3%)		2876 (84.4%)	2742 (75.4%)	
Poorest (Q1)	1200 (32.4%)	1546 (41.7%)		533 (15.6%)	893 (24.6%)	
Nearest Health Facility (km)	2.89 ± 0.54	4.53 ± 0.98	0.005	2.60 ± 0.56	4.24 ± 0.95	0.004

### Statistical analysis

To permit estimation of difference-in-differences (DiD) effects, longitudinal observation of clusters was combined with sampling within baseline clusters for the endline survey. Intra-cluster correlation between children of households in the same enumeration area was accounted for using robust standard errors via the sandwich estimator. With only four treatment districts and seven comparison districts, the number of districts was insufficient to provide a basis for randomization. However, for GEHIP to be relevant to policy makers, units of observation were required that conformed to units of programmatic decision-making represented by the district. In the absence of adequate statistical power at this organizational level, GEHIP embraced a quasi-experimental plausibility design. Owing to the policy relevance of this configuration, such designs have received growing attention in the implementation science literature, building upon the pioneering work of Campbell and Stanley (1966), and more recent advocacy of plausibility designs for implementation research [45–47]. Statistically rigorous responses to plausibility designs have been widely used with inference based on the Heckman difference in difference (DiD) concept [48] for the calculation of average treatment effects based on aggregate data [49]. A regression extension of the DiD concept is estimated for the present analysis that is based on individual observation [50]. In our mortality analysis, the DiD is a ratio of ratios comparing the ratio measuring mortality change in the treatment area over time with the corresponding ratio measuring mortality change in the comparison area over the same time period.

Employing controls for pre- and post- treatment conditions, the GEHIP average treatment effect is estimated using a hazard model in which G_*i*_ is scored 1 if individual child *i* is resident in a GEHIP treatment area household and zero otherwise. P_*it*_ indicates period, where child *i* in month of life *t* is scored 1 if the month of life is July 2011 or after (the post-treatment time period) and zero otherwise. The DiD parameter is the interaction between P and G, **δ**_*ki*_, a parameter representing the net GEHIP incremental effect, relative to trends or areal differences that are unrelated to intervention, while also controlling for the k^th^ maternal or household characteristic of child *i*. The overall GEHIP average treatment effect is given by the conditional hazard:

$$h(t/G,P,x)=h_{0}(t)e^{{\beta G}_{i}+\gamma P_{ij}+\delta P_{ij}G_{i}+\sum_{c=1}^{C}\varphi_{ik}x_{ic}}$$ (1)

**β, γ,** and **δ** are unknown parameters estimated by maximum likelihood. Background characteristics comprising the vector ***x*** comprised of ***C*** household indicators of distance to clinical care facilities and relative household economic status as well as maternal age, parity, educational attainment, and marital status, permitting estimation of K multivariate **δ** “nuisance” parameters that introduce control for imbalance (Table 1). Multiple imputation by chained equations was employed to account for missing values [51]. Greenland’s procedure for achieving model parsimony is used to estimate the final model, which is the reduced form of (1) [52–56]. This procedure introduces parsimony into the estimation of models that could otherwise acquire sparse data biases [54, 57–59]. The final reduced model was identified using Greenland’s recommended modeling strategy that combines a change-in-estimate approach with reduction of mean squared error. The final model excludes covariates included in the full model (1) that do not confound to the effect estimate and which, if removed, reduce the mean squared error of the effect estimate [54].

Ethical approval for the Ghana Essential Health Intervention Project (GEHIP) was granted by Ethical Review Committee of the Ghana Health Service, the Institutional Review Board (IRB) of the Navrongo Health Research Centre and the ethical review board of the Columbia University Medical Center, Mailman School of Public Health. A written inform consent was provided to study participants prior to their inclusion. Data collectors read a written informed consent form to participants in their preferred language and explained its content before participants who agreed to participate endorsed two copies of the form and a copy was given to the participant. This procedure was sanctioned by all three ethics committees that approved of the study to be conducted. All protocols were followed to ensure confidentiality during data collection, analysis and reporting of findings.

## Results

### Statistical balance

Table 1 presents background characteristics of children on key variables across treatment groups. No major imbalances in the shift in baseline versus endline distributions of children by gender across treatment groups, and between baseline and endline between the characteristics of intervention and comparison areas are evident. In terms of age at childbearing, the proportion of younger women giving birth declined during the intervention period. This trend was more pronounced in the intervention arm. With regards to birth spacing, while there were no meaningful differences across treatment groups at both the baseline and endline periods, there were noticeable declines in the percentages of children at endline who were born in less than 24 months between the previous and the subsequent pregnancy. Thus, birth spacing increased among mothers during the intervention period and across treatment groups. While relative poverty decreased over time in both treatment and intervention areas, within each period, a greater proportion of children from the intervention area were in the poorest quintile. These differentials, while minor, attest to the statistical value of the DiD plausibility adjustments estimated by our full multivariate regression model with controls for potential confounders.

### Hazard regression results

**Fig 2:**
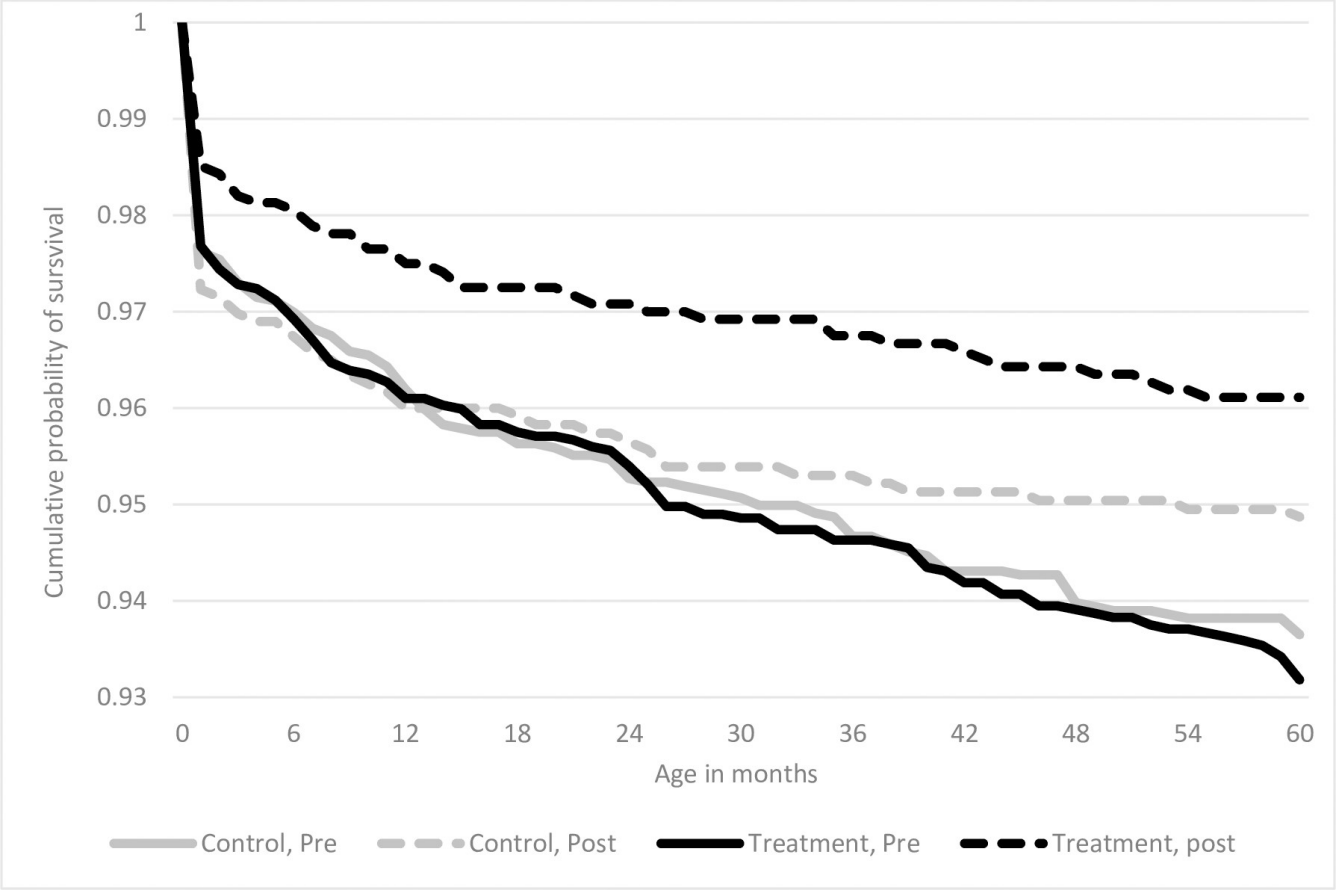
Kaplan-Meier cumulative probabilities of survival by age of under-5 child, treatment area and time period.

Fig 2 presents’ unadjusted Kaplan-Meier survival curves for person-months of observation among all children included in the sample, comparing cumulative survival associated with ever exposure to GEHIP interventions, with corresponding survival for person-months of never exposed during the same pre and post time periods. As Fig 2 shows, survival rates were higher for GEHIP exposed children.

### Multivariate analysis

It is not possible to obtain an overall estimate of mortality for all children under 5 because the mortality hazard ratio varies by age. Treatment area neonatal mortality declined by approximately one third (HR = 0.64, 95% CI = 0.39, 1.05, p = 0.075) between the pre and post periods, while neonatal mortality in the comparison area actually increased over the same time period (HR = 1.22, 96% CI = 0.82.1.81, p = 0.327). Neither of these changes over time were statistically significant. The Difference in Differences estimator for the incremental effect of GEHIP, comparing mortality change in the treatment area with mortality change in the comparison area, indicates that the GEHIP package of interventions reduced neonatal mortality by almost one half (DiD HR = 0.52, 95% CI = 0.28,0.98; p = 0.045) relative to the comparison area (see Final Model in column 3 of Table 2).

**Table 2:** Estimated effect of GEHIP on under age 5 child mortality, Upper East Region, Ghana, 2005–2014.

	Unadjusted Model (1)			Full Model (2)			Final Model (3)		
	HR	95% CI	p-value	HR	95% CI	p-value	HR	95% CI	p-value
**Treatment**									
Comparison area	1.00	ref		1.00	ref		1.00	ref	
Treatment Area (GEHIP Area)	0.97	(0.66–1.42)	0.888	0.96	(0.65–1.41)	0.826	0.97	(0.66–1.42)	0.910
**Period**									
Pre: All person months prior to July 2011 (time before GEHIP began in July 2011)	1.00	Ref		1.00	Ref		1.00	ref	
Post: All person time July 2011 or later	1.17	(0.80–1.71)	0.430	1.22	(0.80–1.86)	0.359	1.22	(0.82–1.81)	0.327
**GEHIP Incremental Effect (DiD)** (Treatment * Post)	**0.55**	**(0.29–1.03)**	**0.064**	**0.53**	**(0.28–1.02)**	**0.056**	0.52*	**(0.28–0.98)**	**0.045**
**Post-neonate infant interaction terms (months 1–12)**									
Treatment * Post-Neonate-Infant	1.14	(0.66–1.96)	0.639	1.13	(0.66–1.92)	0.663	1.13	(0.66–1.93)	0.665
Period * Post-Neonate-Infant	0.74	(0.39–1.40)	0.352	0.73	(0.39–1.38)	0.329	0.72	(0.38–1.37)	0.319
GEHIP DiD * Post-Neonate-Infant	1.34	(0.45–3.99)	0.597	1.40	(0.47–4.20)	0.546	1.38	(0.46–4.12)	0.564
**Post-infant interaction terms (months 12–60)**									
Treatment * Post-Infant	1.18	(0.77–1.80)	0.453	1.15	(0.76–1.73)	0.513	1.16	(0.77–1.77)	0.474
Period * Post-Infant	0.38**	**(0.20–0.71)**	**0.003**	0.41**	**(0.21–0.78)**	**0.007**	0.37**	**(0.20–0.70)**	**0.002**
GEHIP DiD * Post-Infant	1.91	(0.83–4.40)	0.126	1.94	(0.84–4.47)	0.118	1.96	(0.87–4.45)	0.107
**Gender**									
Male				1.00	ref				
Female				0.78**	(0.64–0.94)	0.010			
**Multiple birth**									
Singleton				1.00	ref		1.00	ref	
Multiple				3.16***	(2.05–4.88)	0.000	4.08***	(2.76–6.02)	0.000
**Gestation** ^**a**^									
9 months				1.00	ref				
< 9 months				4.52***	(2.96–6.91)	0.000			
**Birth spacing**									
≥ 24 months				1.00	ref				
< 24 months				1.71***	(1.32–2.21)	0.000			
**Maternal Age**									
15–20				1.00	ref				
20–34				1.07	(0.77–1.49)	0.690			
35–49				1.00	(0.63–1.57)	0.983			
**Parity**									
Nulliparous				1.00	ref				
Primipara				1.03	(0.74–1.45)	0.843			
Multipara				0.74	(0.55–1.01)	0.056			
Grandpara				0.77	(0.52–1.15)	0.205			
**Marital status**									
Unmarried				1.00	ref				
Polygynous				1.08	(0.74–1.56)	0.703			
Monogamous				0.93	(0.68–1.26)	0.627			
**Literacy**									
No				1.00	ref				
Yes				0.98	(0.77–1.24)	0.838			
**Household wealth**									
Less poor (Q2-5) ^**b**^				1.00	ref				
Poorest (Q1)				1.06	(0.82–1.36)	0.657			
**Nearest clinical health facility to household (km)** ^**c**^				1.02	(0.99–1.06)	0.178	1.02	(0.99–1.06)	0.273
	**Summary statistics:**								
Observations (Person-time)	424,909			424,909			424,909		
Number of. Subjects	14,454			14,454			14,454		
Number of Deaths	445			445			445		
Number of Clusters	66			66			66		
F statistic (d.f.)	3.84 (9)			10.75 (23)			8.57 (11)		

* p<0.05

** p<0.01

*** p<0.001

^a^ Self-reported duration of gestation

^b^ Principal component quintile for relative household economic status = 1 for poorest and zero for other quintiles

^c^ Time-varying covariate; distances tabulated for each household to nearest CHPS facility for every month from 2000–2014

**Fig 3:**
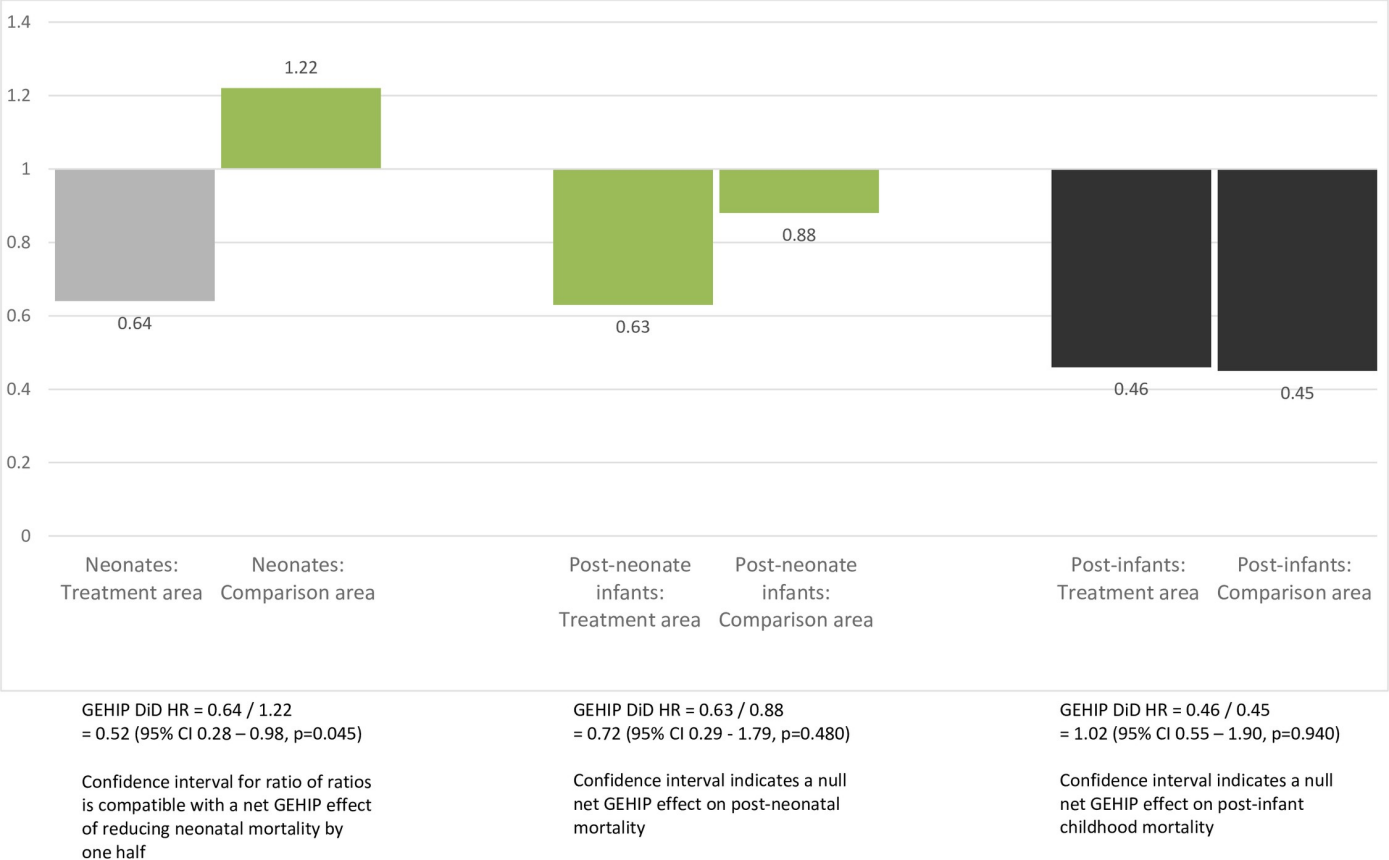
Mortality change over time in treatment and comparison areas: Hazard ratios comparing mortality in the post-treatment period with mortality in the pre-treatment period, by age (from Final Model, Table 2).

Linear combinations of estimators presented in Table 3 are graphed in Fig 3. As the figure shows, in the post-neonatal-infant period, there was no significant change in mortality between pre and post periods in either the treatment or comparison area and a null net effect of GEHIP for this age group. Among post-infants, mortality declined by about one half in both the treatment (HR = 0.46; CI = 0.30,0.71; p = 0.000) and comparison areas (HR = 0.45; CI = 0.29,0.71; p = 0.001). Declines were unrelated to the trial (DiD HR = 1.02; CI = 0.55,1.90; p = 0.940). For post-infants, there was also a null incremental effect of GEHIP since the pronounced decline in mortality was equivalent in the treatment and comparison areas.

**Table 3:** Results from analyses of linear combinations of estimators from Table 2 and Table 3, comparing mortality changes over time in the treatment area with mortality changes in the comparison area.

	Hazard ratios comparing mortality of children in the post-treatment period (July 2011 or later) with mortality of children during the pre-treatment period	Difference in differences (Ratio comparing change in treatment with change in comparison)
**Neonates, adjusted**		HR = 0.52* (0.28, 0.98), p = 0.045
Treatment	HR = 0.64^**¥**^ (0.39, 1.05) p = 0.075	
Comparison	HR = 1.22 (0.82, 1.81), p = 0.327	
**Post-Neonatal Infants, adjusted**		HR = 0.72 (0.29, 1.79), p = 0.480
Treatment	HR = 0.63 (0.29, 1.37), p = 0.247	
Comparison	HR = 0.88 (0.54, 1.43), p = 0.611	
**Post-Infants, adjusted**		HR = 1.02 (0.55, 1.90), p = 0.940
Treatment	HR = 0.46*** (0.30, 0.71), p = 0.000	
Comparison	HR = 0.45** (0.29, 0.71), p = 0.001	

^**¥**^ p<0.10

* p<0.05

** p<0.01

*** p<0.001

Adjustment for background characteristics posited to confound the DiD estimate had no appreciable effect on estimated GEHIP effects. Factors that affect childhood mortality include (1) gender, with females experiencing one quarter lower mortality than males (HR = 0.78, 95% CI 0.64, 0.94, p = 0.010); (2) being a multiple birth, with multiples experiencing three times the mortality of singletons (HR = 3.16, 95% CI 2.05, 4.88, p = 0.000); (3) duration of gestation, with children born prior to nine months gestation experiencing over four times the mortality of children carried to term (HR = 4.52, 95% CI 2.96, 6.91, p = 0.000); and (4) birth spacing, with children both less than two years after their mother’s last birth experiencing almost twice the mortality of those born after two or more years (HR = 1.71, 95% CI 1.32, 2.21, p = 0.000). Other background characteristics, such as mother’s parity, marital status, age and literacy, as well as household socioeconomic status (SES), had no apparent effect on survival.

## Discussion

The results portrayed in the Kaplan-Meier graph illustrate the survival advantage experienced by children who were resident in the treatment areas relative to children in the comparison areas without exposure to GEHIP health systems strengthening interventions. Decomposition of the child survival effect into different under age five groups showed that GEHIP impact was most apparent on neonatal mortality. The neonatal effect may have arisen as a result of the introduction of the emergency referral system that was part of the package of interventions to deal with persistently high neonatal mortality in many of parts of Ghana and elsewhere in Africa. This strategy was actively supported by CHPS outreach activities that were more rigorously implemented in districts where GEHIP was actively promoting CHPS scale-up. However, while the results showed substantial reductions in post-infant mortality during the period of the intervention in UER, the reductions occurred equivalently in the GEHIP exposed and non-exposed areas. This may be due to the effects of implemented interventions in the treatment areas that may have been taken up by the non-intervention area districts even before the trial period ended. Given that the GEHIP intervention was embedded within the health system and implemented by the Ghana Health Service, for ethical reasons training activities designed to improve the coverage of IMCI were instituted in all UER districts, and revenue for primary health care activities in general were allocated to all districts to ensure equivalent access to staffing and pharmaceutical supplies. Moreover, the limited duration of observation of children in late childhood was associated with censoring of observations, constraining prospects that GEHIP could produce definitive inference for post-infancy ages owing to power constraints [60].

Children who were born after the start of GEHIP benefitted from maternal health education at ante-natal care, post-natal care, and perinatal immunization, as well as IMCI services at older ages. These interventions are well understood and supported, necessitating training and intervention in all UER districts, including comparison districts. Although children born before the start of GEHIP in July 2011 could only benefit from GEHIP at older ages, children exposed to GEHIP at birth may have benefited from newborn focused interventions that were not available to children born before the onset of GEHIP operations.

These results are consistent with the possibility that generalized childhood health care promotion and services in all districts of the UER impacted older children in both treatment and comparison areas. [61, 62] Such an explanation is consistent with economic analyses showing that investment of donors, such as UNICEF, expanded the intensity of late childhood focused interventions, such as the package of services known as Integrated Management of Childhood Illnesses (IMCI) [63]. Successful and pronounced systems improvements were instituted in both treatment and comparison districts, balancing the GEHIP-comparison district overall economic investment in primary health care. This policy in the region may have offset prospects that GEHIP could have had an added value among post-infants.

The pronounced impact of GEHIP among neonates, however, merits careful review for policy implications. Several studies of the impact of community-based primary health care on neonates have demonstrated null effects[36, 64–67]. Yet, several studies have demonstrated the potential impact of community-based care for febrile illnesses (Bhutta et al. 2009; Zaidi et al. 2011). Although some studies have demonstrated strategies that are likely to be feasible to implement at scale, all community-based studies demonstrating neonatal effects, other than an investigation in Tanzania [66], have been based in Asia [67–69].

No single intervention explains the GEHIP success. Comprised of a combination of leadership and community support for outreach and care, emergency referral services, and expanded coverage of primary care services, GEHIP demonstrated a strategy for saving newborn lives.

### Limitations

Retrospective assessment of child survival via birth history analysis may be subject to event omission or event displacement recall biases [70]. Moreover, the trauma or stigma of losing a child may lead mothers to omit the reporting of some deaths [71]. Neonatal mortality may be particularly under-reported if newborn deaths are selectively omitted or misreported as stillbirths [72]. All analyses are predicated on the assumption that such recall biases apply equivalently to GEHIP exposed and unexposed respondents. Moreover, controls for source of data are posited to offset any such bias. By sampling living women of reproductive age, analyses would omit the survival experience of children with deceased mothers. If GEHIP interventions have affected maternal mortality, this would introduce a bias in the present analysis.

Any plausibility trial of health systems development is embedded in routine operations of the host system environment. The hierarchical organizational structure of the health delivery model of the Ghanaian system is potentially comprised of health posts at the community level, sub-district clinics and district level hospitals. Although primary health care operations were focused on services at the community level, the leadership, supervisory, and resource interventions of GEHIP aimed to develop district support systems that could benefit the functioning of community health nurses at the periphery of the hierarchy. This hierarchical interdependence introduced an element of complexity which statistical models in this analysis may not have fully addressed. Although the lack of a rigorous specification of counterfactual conditions is addressed, in part, with the Heckman difference-in-differences approach, routine administrative decisions selectively imposed during the intervening period could have compromised design balance. In response to this challenge, GEHIP interventions were complex, both in terms of the deployment of the interventions and the modeling of a multifaceted but coordinated series of interlocking interventions at different levels at different points in time. Because children were first exposed to GEHIP interventions at different ages, analyses were designed to account for changing childhood exposure to systems change as GEHIP progressed. The GEHIP endpoint, under five mortality, was analyzed for all person-months of observation of children who were ever aged 60 months or less during the study. Specification within a DiD framework allowed the mortality of treatment and comparison area children to be measured and compared during the “pre” treatment period prior to July 2011 and during the “post” period beginning in July 2011. Age interaction terms ensured that exact age conditionality of posited impact was addressed.

## Conclusion

Evaluations of community health programs in Africa have repeatedly shown that childhood mortality impact of community-based care is most pronounced among post-infant children who are vulnerable to acute respiratory infections, malaria, and diarrheal diseases, and responsive to the range of care that community health workers can effectively purvey [73]. The impact of such programs on the survival of newborns is typically less pronounced or even absent [74, 75], largely because the impact of infectious disease on the survival of neonates is less problematic than non-infectious disease causes of morbidity and mortality that are directly related to birth and the need for immediate postpartum care [65, 76, 77].

The success of GEHIP among neonates, with its package of community-engaged approaches to sustaining emergency referral and providing doorstep post-delivery surveillance, is therefore directly relevant to policy. GEHIP results show that a comprehensive approach to newborn care is feasible, even if care is augmented by community-based nurses. Mobilizing community action to promote facility-based delivery, with support for essential logistics services, set the stage for GEHIP impact. However, retraining of workers to conduct post-delivery household visits and care for asphyxia, febrile illnesses, and recognition of emergency needs was also important. Of equivalent importance was the building of a system of sustainable logistics support whereby volunteers managed, operated and maintained low cost transportation equipment. This system of frontline care was supported by a corresponding system of community, political, and leadership engagement to marshal resources and sustain primary health care governance. The result was an acceleration of community service functioning and an intensification of access to care that has saved child lives. The task ahead requires policy and action to scale-up GEHIP in the UER and test feasible means of replicating project activities in other regions as a national program of CHPS reform.

## Supporting information

S1 Table (DOCX)

## Abbreviations

ANC: Antenatal care
CHPS: Community Health Planning and Services Initiative
DHS: Demographic and Health Survey
GEHIP: Ghana Essential Health Interventions Programme
GHS: Ghana Health Service
HR: Hazard ratio
IMCI: Integrated management of childhood illness
PCA: Principal Components Analysis
PNC: Postnatal care
UER: Upper East Region
WHO: World Health Organization
