# Supplementary material for: The child survival impact of the Ghana Essential Health Interventions Program: A health systems strengthening plausibility trial in Northern Ghana
Source: PLoS One. 2019 Jun 12;14(6):e0218025. doi: 10.1371/journal.pone.0218025 (PMC6561634; doi:10.1371/journal.pone.0218025)
Supplement: S1 Table — (DOCX) [file pone.0218025.s001.docx]

**Table S1**: The operational problems that were addressed with particular interventions under GEHIP

| **GEHIP Interventions** | | |
| --- | --- | --- |
| **Organizational level of intervention** | **Type of operational problem to be addressed** | **Intervention** |
| **District Health Management Team** | Lack of strategic understanding of the CHPS model | Field exchanges and demonstration added to routine leadership training workshops |
|  | Lack of flexible funding | Addition of $0.85 per capita per year for three years to the District Common Fund. |
|  | Lack of evidence-based planning and budgeting | Provision of a burden of disease-based planning tool. |
| **District Hospital and Sub-district Health Centers clinical services** | - Excess neonatal mortality - Excess maternal morbidity and mortality | - Post-natal care for newborns (KMC, other interventions) - Directly Observed Therapy, Short-course (DOTS) - Emergency management: Comprehensive Emergency obstetric services to include basic Emergency obstetric care, comprehensive Emergency obstetric services, case management of newborn illnesses, comprehensive emergency referral logistics equipment, and newborn resuscitation training |
| **Sub-district Health Centers clinical services** | Lack of supervisory support for frontline providers | - Facilitative supervision training - Information for decision-making systems reform |
| **CHPS level interventions** | Lack of functioning information systems  Lack of field work, community outreach, doorstep services  Lack of organized program of referral, triage, and community engagement for emergency public health | Simplification and reform of clinical recording systems  Supervisory support for community-based care.  Comprehensive training in emergency management to include:   - Newborn surveillance and care, including resuscitation training, febrile illness recognition, and other immediate newborn monitoring - Emergency communication and referral equipment and support. - Motorcycle ambulance provision. - Volunteer ambulance driver training and deployment - Community outreach training |
